# Production of a Subunit Vaccine Candidate against Porcine Post-Weaning Diarrhea in High-Biomass Transplastomic Tobacco

**DOI:** 10.1371/journal.pone.0042405

**Published:** 2012-08-03

**Authors:** Igor Kolotilin, Angelo Kaldis, Bert Devriendt, Jussi Joensuu, Eric Cox, Rima Menassa

**Affiliations:** 1 Southern Crop Protection and Food Research Centre, Agriculture and Agri-Food Canada, London, Ontario, Canada; 2 Laboratory of Veterinary Immunology, Faculty of Veterinary Medicine, Gent University, Merelbeke, Belgium; 3 VTT Technical Research Centre of Finland, Espoo, Finland; University of Padova, Italy

## Abstract

Post-weaning diarrhea (PWD) in piglets is a major problem in piggeries worldwide and results in severe economic losses. Infection with Enterotoxigenic *Escherichia coli* (ETEC) is the key culprit for the PWD disease. F4 fimbriae of ETEC are highly stable proteinaceous polymers, mainly composed of the major structural subunit FaeG, with a capacity to evoke mucosal immune responses, thus demonstrating a potential to act as an oral vaccine against ETEC-induced porcine PWD. In this study we used a transplastomic approach in tobacco to produce a recombinant variant of the FaeG protein, rFaeG_ntd/dsc_, engineered for expression as a stable monomer by N-terminal deletion and donor strand-complementation (ntd/dsc). The generated transplastomic tobacco plants accumulated up to 2.0 g rFaeG_ntd/dsc_ per 1 kg fresh leaf tissue (more than 1% of dry leaf tissue) and showed normal phenotype indistinguishable from wild type untransformed plants. We determined that chloroplast-produced rFaeG_ntd/dsc_ protein retained the key properties of an oral vaccine, i.e. binding to porcine intestinal F4 receptors (F4R), and inhibition of the F4-possessing (F4+) ETEC attachment to F4R. Additionally, the plant biomass matrix was shown to delay degradation of the chloroplast-produced rFaeG_ntd/dsc_ in gastrointestinal conditions, demonstrating a potential to function as a shelter-vehicle for vaccine delivery. These results suggest that transplastomic plants expressing the rFaeG_ntd/dsc_ protein could be used for production and, possibly, delivery of an oral vaccine against porcine F4+ ETEC infections. Our findings therefore present a feasible approach for developing an oral vaccination strategy against porcine PWD.

## Introduction

Enterotoxigenic *Escherichia coli* (ETEC) strains that produce long proteinaceous appendages on their surfaces, called F4 fimbriae (F4+ ETEC), are the key culprit for Post-Weaning Diarrhea (PWD) among newly weaned piglets worldwide, which results in morbidity, reduced growth and mortality, causing severe economic losses. These ETEC strains are often associated with multiresistance to several antimicrobials probably caused by the prophylactic use of antibiotics [1], [2]. Following deprivation of passive lactogenic immunity from parenterally vaccinated sows, the small intestine in newly weaned piglets becomes the main gateway for invading pathogenic F4+ ETEC, which infect, colonize and produce enterotoxins, changing the water and electrolyte flux of the small intestine and leading to PWD, weight loss and often death [3], [4]. Vaccination of weaned piglets would be a desirable means of controlling ETEC-induced PWD; however, an effective vaccine against porcine PWD, which is cheap to produce and administer, is currently unavailable. Injectable vaccines, such as those administered to sows are expensive and tend to stimulate systemic rather than protective mucosal immune responses needed to prevent intestinal ETEC infection [5].

Encoded by the *fae* operon, F4 fimbriae are polymers, composed mainly of several hundreds of identical protein subunits called FaeG, as well as minor subunits, such as FaeC, FaeF, FaeH and FaeD [6], [7]. The periplasmic chaperone FaeE plays a crucial role in F4 fimbriae assembly, which occurs through a donor strand complementation/exchange mechanism [8], [9]. Initially, FaeE interacts with the C-terminal part of FaeG and complements its folding with a chaperone donor β-sheet, following which the donated β-sheet is replaced by an N-terminal β-sheet of another FaeG subunit. This completes the folding of each subunit and connects the subunits to each other to form the polymeric F4 fimbriae structure [8]. Three serological variants of F4 fimbriae, namely F4_ab_, F4_ac_ and F4_ad_ have been identified by differences in the sequence of the major subunit FaeG, which contains conserved regions designated “a” and variable regions forming “b”, “c”, and “d” determinants [9]–[14].

The F4 fimbrial adhesin FaeG mediates F4+ ETEC adherence to F4-specific receptors (F4R) on small intestinal enterocytes, thus initiating a primary and essential step for infection [15]–[18]. Being an important F4+ ETEC virulence factor, the FaeG protein was shown to possess strong antigenic properties, and was identified as a prospective candidate for the development of an oral subunit vaccine against F4+ ETEC infections [19]–[23]. Oral vaccination of piglets with recombinantly-produced FaeG induced F4-specific systemic and mucosal immune responses [5], [21], [23], [24].

The feasibility of production of a functional recombinant (r) FaeG protein has been investigated in bacteria [23], [24] and in plants [20], [21], [25]–[27]. Initial studies in *E. coli* showed that rFaeG was found in an insoluble and inactive form in inclusion bodies, and laborious re-folding procedures were required for production of a conformational rFaeG structure similar to that in native F4 fimbriae, yet much less stable [23], [24]. On the other hand, nuclear-transformed tobacco plants, expressing rFaeG targeted to different sub-cellular compartments, demonstrated that the chloroplast was a superior environment for accumulation of a soluble and stable form of rFaeG, which reached 1% of total soluble proteins (TSP) [25], [28]. Structural characterization of the chloroplast-targeted rFaeG protein revealed a unique spontaneous assembly of the rFaeG protein monomers into strand-swapped dimers, in which the monomers mutually complemented each other’s fold, conferring its stability and suggesting existence of a chloroplast-residing FaeE-like chaperone [28]. Based on the crystallized structure of the chloroplast-accumulated rFaeG dimers, an N-terminal-deleted (ntd), donor-strand-complemented (dsc) monomeric rFaeG (rFaeG_ntd/dsc_) was designed. In rFaeG_ntd/dsc_ the N-terminal domain, which is involved in donor strand exchange between native FaeG subunits during fimbriae assembly was fused to the FaeG C-terminus through a linker, allowing it to fold back and stabilize the core FaeG, resulting in a soluble and stable monomeric structure [8]. Although the structural and biophysical properties of rFaeG_ntd/dsc_ were extensively characterized [8], the capacity of this engineered FaeG variant to express to high levels in plants and serve as an oral subunit vaccine against F4+ ETEC remains unknown.

Plant-produced subunit vaccines present a safer choice than the conventional recombinant production systems, such as bacteria, yeast or mammalian cells, since contamination risk with mammalian pathogens and/or endotoxins is minimized. High safety standards of plants as bio-factories are coupled with low production and delivery costs and ease of scale-up, which makes plants a preferable recombinant production platform [29]–[34]. Further, plants with a transformed plastid genome (plastome) have persistently demonstrated capability to produce very high yields of various foreign proteins, reaching 20–40% TSP in leaf tissue [35]–[39]; for review see [40]–[43]. In comparison with classical nuclear transformation, plastome engineering is considered to have several advantages, such as lack of positional effects or transgene silencing. Plastomes are nearly exclusively maternally transmitted, providing almost perfect biological containment for the engineered genetic material [44], [45]. Chloroplast-expressed proteins are not glycosylated, eliminating the possibility of addition of potentially allergenic non-mammalian glycans to recombinant proteins; this feature makes transplastomic technology particularly favourable for expression of non-glycosylated proteins of prokaryotic origin [46], [47]. Indeed, successful and prolific expression of vaccine antigens in engineered chloroplasts has been reported in numerous studies (for review see [42], [48], [49]).

In the present study we report the high level production of the rFaeG_ntd/dsc_ protein in transplastomic tobacco plants as well as *in vitro* characterization of its vaccine properties. Cumulatively, our results support the development of rFaeG_ntd/dsc_ as a protective oral subunit vaccine against F4+ ETEC, as well as underline that transplastomic tobacco is a very efficient platform for rFaeG_ntd/dsc_ production.

## Results and Discussion

### Chloroplasts can Accumulate High Levels of rFaeG_ntd/dsc_

**Figure 1:**
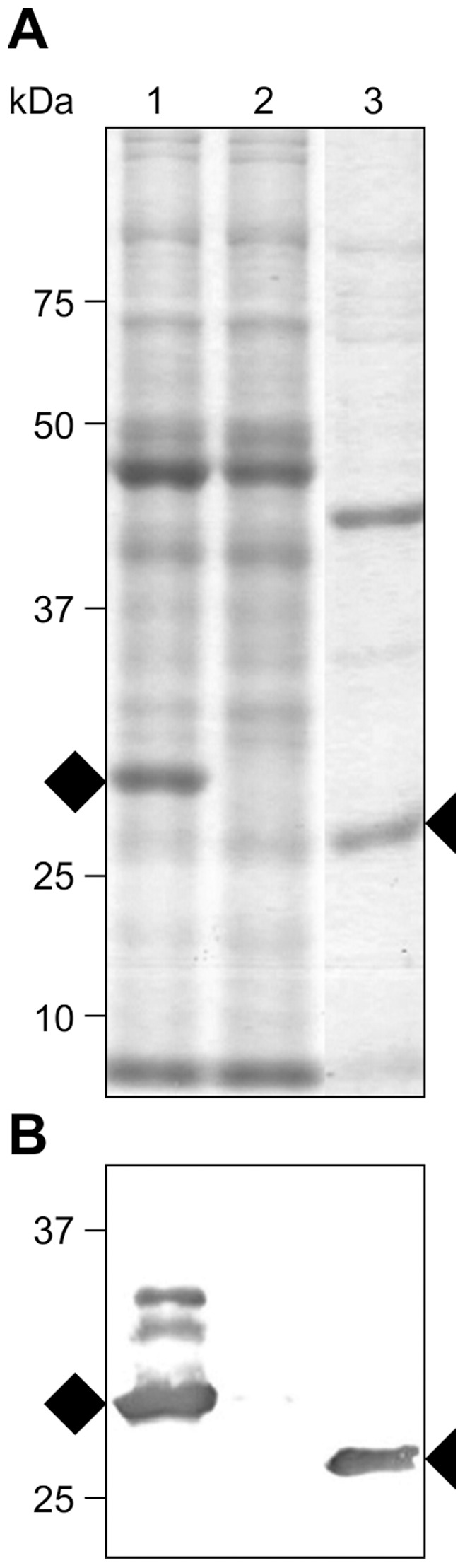
Accumulation of chloroplast-targeted, transiently-expressed rFaeG_ntd/dsc_. Transient expression of the rFaeG_ntd/dsc_ protein via agroinfiltration in *Nicotiana benthamiana* leaves was examined by SDS-PAGE and staining (a), and immunoblot analysis (b). Lanes 1 and 2−5.0 µg of protein extract of leaves co-infiltrated with *Agrobacteria* carrying chloroplast-targeted rFaeG_ntd/dsc_ and the p19 viral suppressor of post-transcriptional gene silencing (1), or p19 alone as negative control (2). rFaeG_ntd/dsc_ is indicated with a black rhomb, higher bands likely correspond to rFaeG_ntd/dsc_ with partially cleaved transit peptide; Lane 3−0.5 µg purified F4_ad_ fimbriae as positive control, the F4 native FaeG is indicated with a black triangle; the ∼2 kDa difference in size of rFaeG_ntd/dsc_ (29 kDa) and the native FaeG (27 kDa) is due to the additional complementing fused domain.

Chloroplast-targeted dimeric rFaeG accumulation reached 1% TSP in nuclear-transformed tobacco and alfalfa plants [21], [28]. Recently reported transplastomic tobacco plants expressed rFaeG only up to 0.15% TSP [27], pointing out possible limitations of tobacco chloroplasts as a sequestration compartment for higher rFaeG yields. To test whether chloroplasts have the capacity to accumulate larger amounts of the monomeric variant rFaeG_ntd/dsc_ [8], we utilized the speed and convenience of transient expression via agroinfiltration in *Nicotiana benthamiana* leaves. Transient expression, coupled with suppressors of post-transcriptional gene silencing usually yields high accumulation levels of recombinant proteins [50], [51]. The results showed that transiently-expressed, chloroplast-targeted rFaeG_ntd/dsc_ accumulated up to ∼15–20% TSP (Fig. 1), thus demonstrating the potential of chloroplasts to accumulate high levels of the rFaeG protein. Additionally, areas in leaves agroinfiltrated with the rFaeG_ntd/dsc_-expressing construct did not show any signs of necrosis, resembling in appearance areas of leaves infiltrated with the control construct expressing the p19 suppressor of posttranscriptional gene silencing alone, unlike our previous results with GFP targeted to the ER, which exhibited complete necrosis of the infiltrated area [52]. These results suggest that high-level accumulation of rFaeG_ntd/dsc_ in chloroplasts is not harmful to the leaf tissue.

### Plastid Transformation Construct Design and Production of Transplastomic Tobacco Plants Expressing the Recombinant Adhesin rFaeG_ntd/dsc_

**Figure 2:**
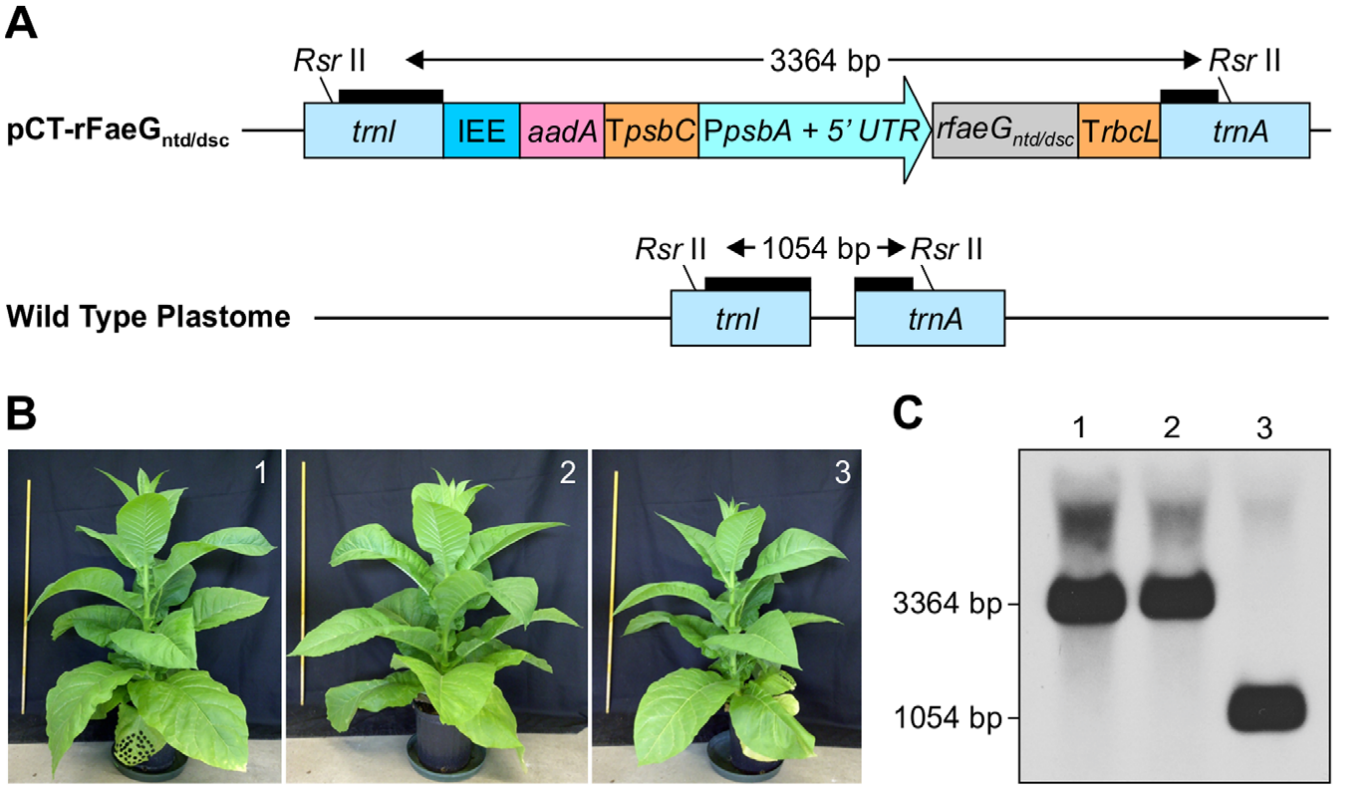
Homoplastomic lines show normal phenotype. (a) A schematic representation of the chloroplast transformation cassette (pCT-rFaeG_ntd/dsc_). The cassette was designed to integrate between the *trnI (tRNA-Ile)* and *trnA (tRNA-Ala)* genes of the tobacco plastome. The wild type (WT) plastome *trnI - trnA* region is shown at the bottom. Expected sizes of *Rsr* II-digested fragments are indicated. Thick black lines represent hybridization sites for the probe used in Southern blot analyses. IEE  =  intercistronic expression element with the Shine-Dalgarno sequence from the 5′ UTR of bacteriophage T7 gene 10 fused to the 3′ end; aadA  =  gene encoding aminoglycoside 3′ adenylyltransferase for spectinomycin resistance; T*psbC*  = 3′ UTR of *psbC* from white poplar plastome; P*psbA*  = 5′ UTR and promoter of tobacco *psbA* gene. *rfaeG_ntd/dsc_*  =  gene encoding the rFaeG_ntd/dsc_ protein variant. T*rbcL*  = 3′ UTR of *rbcL* from white poplar plastome. (b) Phenotypes of mature transplastomic tobacco cv. I 64 plants transformed with pCT-rFaeG_ntd/dsc_ (1 and 2) were indistinguishable from WT plants (3). A one-meter ruler was photographed to the left of each plant as size reference. (c) Confirmation of homoplastomy. Southern blot analysis of total plant DNA from 2 independent transformants and 1 untransformed plant displayed a single band of the expected size.

Numerous viral and bacterial antigens have been expressed in chloroplasts with levels of expression varying from 0.002% TSP [53] to 72% total leaf proteins [54]; reviewed in detail by [42]. Because we found that chloroplast-targeted FaeG_ntd/dsc_ can accumulate to high levels transiently, we decided to express it from the tobacco chloroplast genome. The chloroplast transformation cassette of the pCT-rFaeG_ntd/dsc_ construct (Fig. 2a) was designed to minimize the use of endogenous tobacco regulatory elements, therefore eliminating the possibility of foreign gene loss through deleterious homologous recombination between the duplicated sequences in the transformed plastome [55]. For that, only two tobacco endogenous cis-acting elements were utilized in the cassette: the chloroplast promoter of the *psbA* gene (P*psbA*) along with its 5′ UTR was used for expression of the *rfaeG_ntd/dsc_* gene; and the intercistronic expression element (IEE), shown to facilitate efficient processing of polycistronic mRNAs [56], was placed upstream of the *aadA* gene. The transformation cassette was integrated into the tobacco plastome between the *tRNA-isoleucine* (*trnI*) and *tRNA-alanine* (*trnA*) genes, a transcriptionally-active spacer region which is transcribed as a part of the *rrn* operon from a strong promoter (P*rrn*) [57]. Read-through transcription from the endogenous P*rrn* was exploited for expression of the *aadA* gene, conferring spectinomycin resistance to transformed chloroplasts. Finally, to stabilize nascent transcripts and prevent degradation by plastid 3′ nucleases, the open reading frames of *aadA* and *rfaeG_ntd/dsc_* were fused with heterologous 3′ UTRs with poor homology to tobacco plastome sequences (Fig. 2a). Hence, our tobacco chloroplast transformation cassette was designed to produce separate monocistronic mRNAs, differing in that way from the construct for transplastomic expression of rFaeG described by another group, where the *aadA-faeG* genes, arranged as an operon in that order, were transcribed from one promoter as dicistronic mRNA [27].

We recently identified *Nicotiana tabacum* cultivar I 64 as the most effective for transiently-expressed recombinant protein production [58]. Additional characteristics, such as high biomass and relatively low alkaloid levels, make cv. I 64 a valuable low-cost, efficient and practical delivery vehicle for an oral vaccine that can stimulate mucosal immunity in the intestine of animals. To our knowledge, there are no reports on chloroplast transformation in *N*. *tabacum* cv. I 64, hence it was of particular interest to obtain and characterize transplastomic cv. I 64 plants expressing the rFaeG_ntd/dsc_ protein.

Transplastomic tobacco cv. I 64 plants were obtained by biolistic delivery of pCT- rFaeG_ntd/dsc_ (Fig. 2a). Regenerated transplastomic plants showed a phenotype identical to wild type (WT) and were fertile (Fig. 2b). Homoplastomy of these clones was confirmed by a Southern blot, which displayed specific binding of the probe to bands of predicted size for transformed and WT untransformed plastid DNA, showing complete absence of WT plastome copies in the transplastomic lanes (Fig. 2c). A higher molecular weight signal was apparent in all three lanes, probably caused by partially digested ctDNA. We observed very high transformation frequencies, generating 14 independent transplastomic clones after bombardment of 3 tobacco cv. I 64 leaves. Using the same transformation construct, we found comparable transformation rates (15 transplastomic clones from 5 bombarded leaves) in our low alkaloid *N*. *tabacum* cv. 81V9 [59]. This is an important finding, given the limited number of published reports on successful chloroplast transformation in tobacco varieties other than the small variety Petite Havana and considerable recalcitrance of some tobacco varieties to chloroplast transformation [60]–[62].

**Figure 3:**
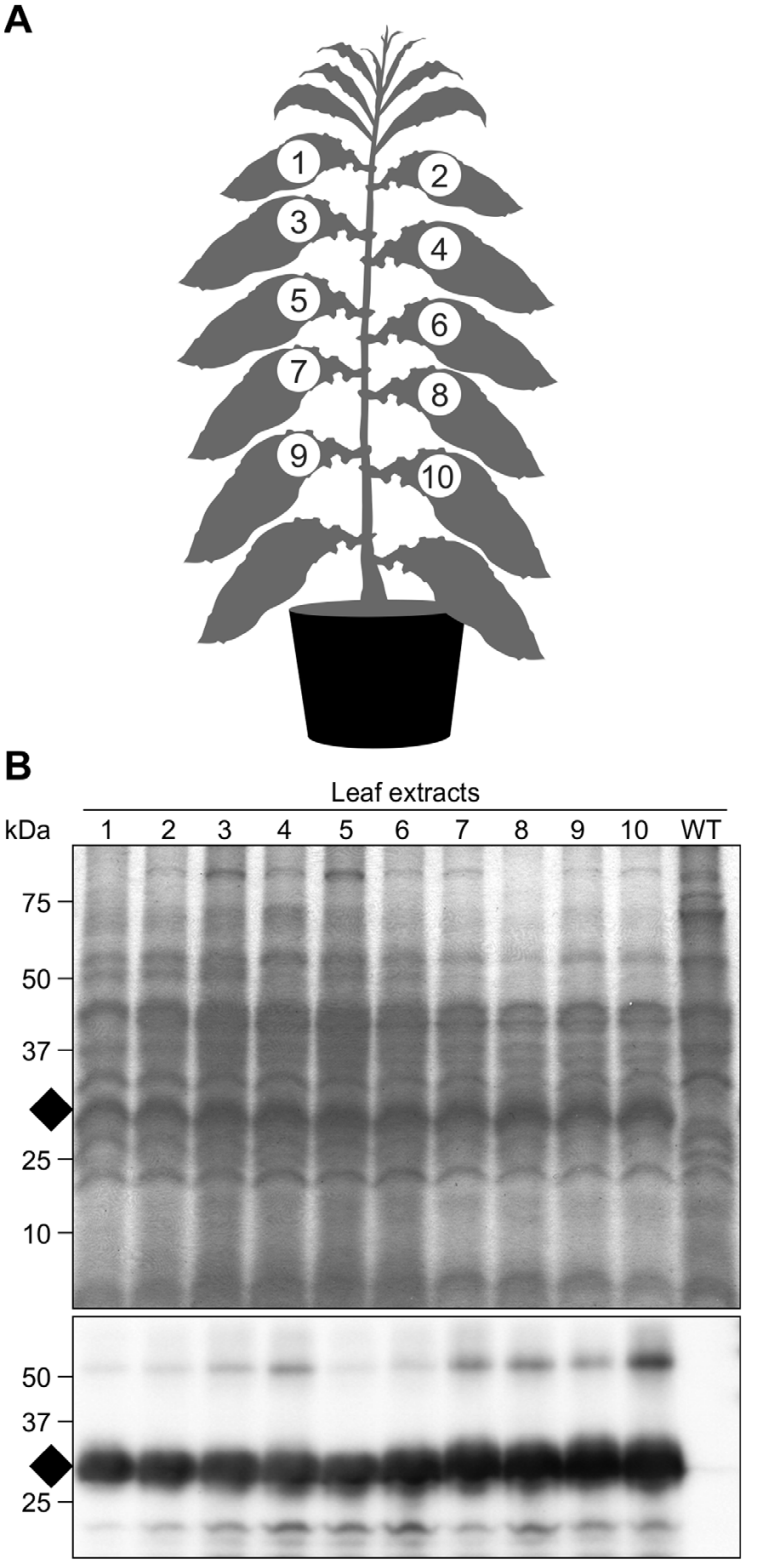
Spatial accumulation of rFaeG_ntd/dsc_ in transplastomic tobacco plants. (a) Schematic showing the 10 leaves sampled to assess the spatial accumulation of rFaeG_ntd/dsc_ in transplastomic tobacco plants. (b) Samples examined on SDS-PAGE stained gel (upper panel) and western blot (lower panel). Each lane was loaded with an extract from either ∼2.3 mg of leaf tissue (stained gel), or ∼0.5 mg (immunoblotted gel). WT =  leaf 4 from an untransformed plant. A band of the predicted size (29 kDa, indicated with a black rhomb) corresponding to rFaeG_ntd/dsc_ was observed in all transplastomic leaf samples, but was absent in the WT. This band was immunoreactive with anti-FaeG serum on the Western blot. kDa - protein molecular weight marker.

To acquire insight into the spatial accumulation pattern of rFaeG_ntd/dsc_ in the whole plant, transplastomic clones were examined for rFaeG_ntd/dsc_ expression before flowering. Samples were taken from 10 leaves, top to bottom (Fig. 3a); proteins were extracted in buffer EB1 at pH 4.9, separated by SDS-PAGE and the gels were stained or immunoblotted (Fig. 3b). Buffer EB1 was used because RuBisCo and other proteins precipitate at that pH while rFaeG_ntd/dsc_ does not. Therefore, the recombinant protein would be easier to visualize in case expression levels are not very high. We found that a band corresponding to rFaeG_ntd/dsc_ was clearly visible in all samples in the stained gel; this band was also immunoreactive with anti-FaeG serum, confirming accumulation of rFaeG_ntd/dsc_ in young as well as in old leaves (Fig. 3b). It’s worthy to notice that accumulation of rFaeG_ntd/dsc_ appeared to be slightly higher in old leaves than in young leaves, whereas the amount of plant endogenous proteins diminished (Fig. 3b). This observation suggests continuous accumulation and stability of the rFaeG_ntd/dsc_ protein inside chloroplasts throughout plant development, probably due to the unique donor strand complementation structure of the rFaeG_ntd/dsc_ monomers [8]. We also observed a less abundant band of ∼58 kDa on the immunoblot, likely corresponding to dimerized rFaeG_ntd/dsc_ (Fig. 3b, lower panel). Formation of strand-swapped dimers of rFaeG_ntd/dsc_ could bring about a stabilizing effect on the protein; this was described for a different chloroplast-targeted rFaeG variant expressed in tobacco nuclear transformants [28].

### Purification and Yield of rFaeG_ntd/dsc_

**Figure 4:**
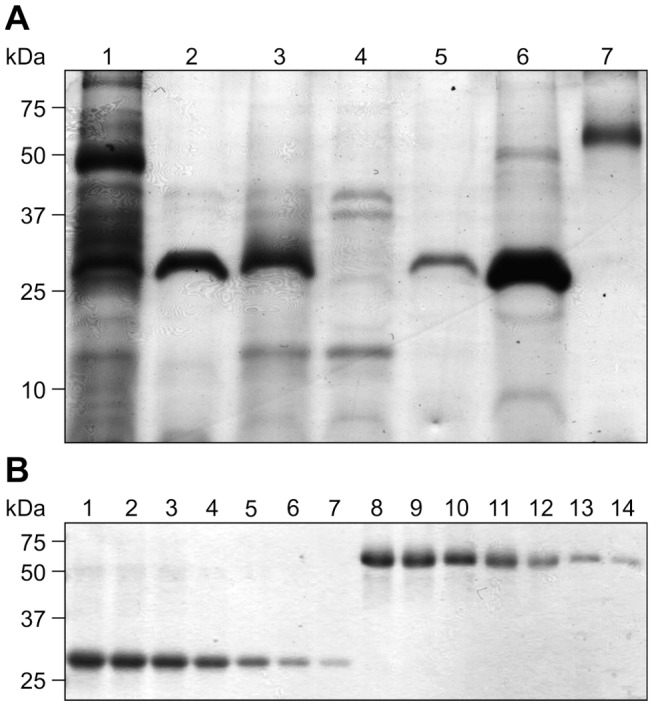
Purification of rFaeG_ntd/dsc_ from crude plant extract and quantification. (a) rFaeG_ntd/dsc_ was extracted from 5 g of mature transplastomic leaf tissue and purified. The initial volume of the extract was 50 ml; 3 µl of the extract from each step of the procedure were resolved by SDS-PAGE and stained. Lane 1 - Initial extract from leaf tissue, pH = 7.5; lane 2 - extract acidified to pH = 2 and centrifuged; lane 3 - clarified extract neutralized to pH = 7.4; Lane 4 - flowthrough from IMAC column; Lane 5 - wash with 20 mM imidazole; Lane 6 - elution of purified rFaeG_ntd/dsc_; Lane 7 - 0.5 µg of BSA as loading control; kDa - protein molecular weight marker. (b) Purified rFaeG_ntd/dsc_ was quantified using densitometry. Dilutions of the purified rFaeG_ntd/dsc_ protein (lanes 1 through 7) were resolved in SDS-PAGE gel along with known amounts of BSA (lanes 8–14; 1.0, 0.8, 0.6, 0.4, 0.2, 0.1, 0.05 µg BSA, respectively) and stained. BSA bands were used for generation of a standard curve (*R^2^* = 0.987; *p* = 0.01) and extrapolating rFaeG_ntd/dsc_ concentration. kDa - molecular weight marker.

After confirming expression of rFaeG_ntd/dsc_ in transplastomic clones, we purified chloroplast-produced rFaeG_ntd/dsc_ and used it as a positive quantifiable control for quantification of rFaeG_ntd/dsc_ yield in transplastomic plants. Since the majority of plant proteins are insoluble at pH<5.0, while the rFaeG protein remains soluble and stable [26], [63], we acidified the extract to pH = 2.0, causing most plant proteins to precipitate. The rFaeG_ntd/dsc_ protein in the clarified extract was then purified by immobilized metal ion affinity chromatography (IMAC), utilizing the N-terminal His-tag fusion (Fig. 4a). The concentration of purified rFaeG_ntd/dsc_ was assessed by comparison with known amounts of bovine serum albumin (BSA) using densitometry (Fig. 4b).

**Figure 5:**
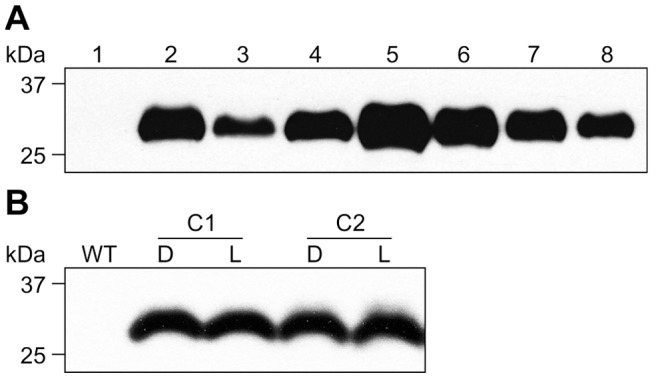
Accumulation levels of rFaeG_ntd/dsc_ in transplastomic leaf tissue. (a) Samples of equal volume (4 µl) were prepared from crude extract fractions. Lane 1 - WT extract (negative control); lanes 2, 3 and 4 represent crude extract of 0.4 mg of leaf tissue, re-extracted pellet, and clarified extract, respectively, where clarified extract contains 5 µg TSP. The rFaeG_ntd/dsc_ yield was estimated using a standard curve (*R^2^* = 0.993) of known amounts of purified rFaeG_ntd/dsc_ (lanes 5 through 8∶2 µg, 1 µg, 0.5 µg and 0.25 µg, respectively). (b) No variation in rFaeG_ntd/dsc_ accumulation was observed in transplastomic clones (C1, C2) after dark (D) or after light (L) periods. Image is representative of sampling on three different days, 1 µg TSP was used per lane. WT =  untransformed control.

Because our goal in the near future is the oral administration of leaves containing rFaeG_ntd/dsc_ to weaned piglets, precise quantification of rFaeG_ntd/dsc_ accumulation in transplastomic leaves is essential for delivery of standardized vaccine doses to animals. Therefore, we determined the accumulation of rFaeG_ntd/dsc_ per leaf fresh weight and dry weight. For this, we homogenized fresh leaf tissue in 10 volumes of extraction buffer and used this crude homogenate for determining the amount of rFaeG_ntd/dsc_ (Fig. 5a, lane 2). To verify if any rFaeG_ntd/dsc_ was trapped in insoluble debris, the crude homogenate was centrifuged; the TSP-containing supernatant was removed and the pellet was re-extracted with an equal volume of extraction buffer. Equal volumes of crude homogenate (Fig. 5a lane 2), supernatant (Fig. 5a, lane 4), and re-extracted pellet (Fig. 5a, lane 3) were separated by SDS-PAGE and analyzed by western blotting. When compared with known amounts of purified rFaeG_ntd/dsc_ (Fig. 5a, lanes 5–8), densitometry indicated that 0.2 mg of rFaeG_ntd/dsc_ is present in 0.1 g of leaf tissue, that about 25% of the rFaeG_ntd/dsc_ is trapped in cell debris, and that rFaeG_ntd/dsc_ represents 11.3% TSP of the first supernatant (Fig. 5a, lane 4). Upon extraction of freeze-dried leaf tissue, we found that rFaeG_ntd/dsc_ constituted 1% of dry leaf weight and 11.3% of TSP, indicating that rFaeG_ntd/dsc_ is stable in dried leaves. The prolific expression of rFaeG_ntd/dsc_ in the generated transplastomic plants suggests that transient expression coupled with chloroplast targeting can be an effective tool for rapid evaluation of the potential of a protein to be successfully expressed in chloroplasts via engineered plastome, even though actual expression levels cannot be predicted.

Our result represents more than a 75-fold increase in the expression levels of rFaeG adhesin compared with previously reported transplastomic tobacco plants expressing a different rFaeG variant [27]. Our construct was designed to express rFaeG_ntd/dsc_ from the *psbA* gene promoter and 5′UTR (P*psbA*), while [27] arranged their construct as an operon *aadA*-*rfaeG* transcribed as dicistronic mRNA. Although in some cases, a similar operon structure resulted in high yields of foreign proteins [64]–[66], a certain bias was demonstrated in the preference of the plastid translation machinery toward predominant utilization of the 5′-most Shine-Dalgarno (SD) sequences on polycistronic mRNAs, while recognition of internal SD sequences is inefficient [67]. Interestingly, expression of human serum albumin (HSA) from a construct built as an operon *aadA*-*HSA* resulted only in 0.02% HSA of total leaf protein, whereas a 360-fold increase in HSA accumulation was observed when the *HSA* gene was placed under the control of the P*psbA* and its 5′ UTR region [68]. In that study, differences in *HSA* mRNA steady state levels could not account for such a boost in HSA expression, suggesting that the 5′ UTR of the *psbA* gene was associated with strong enhancement of translation; this is supported by similar findings from other studies [69]–[71]. Our results confirm the idea that the *psbA* 5′ UTR mediates efficient translation of the rFaeG_ntd/dsc_-encoding transcript which at least partly explains our high levels of rFaeG_ntd/dsc_ compared to the work of [27]. Another factor that could account for high rFaeG_ntd/dsc_ accumulation is the structural specificity of the variant we used, which was engineered to have a complementing donor strand previously reported to stabilize rFaeG_ntd/dsc_ in its monomeric soluble form [8].

Because translation of rFaeG_ntd/dsc_ is controlled by the 5′ UTR region of the *psbA* gene, which was reported to be induced by light [70], [71], we compared levels of rFaeG_ntd/dsc_ in the leaves of two greenhouse-grown transplastomic clones, harvested before sunrise and before sunset of a sunny day on three different days. Analysis of the collected samples did not reveal any diurnal variation in the levels of rFaeG_ntd/dsc_ accumulation (Fig. 5b), suggesting a very low rate of foreign protein turnover in chloroplasts, which is supported by the observation of higher rFaeG_ntd/dsc_ levels in older leaves (Figure 3b). Although some studies that utilized P*psbA* 5′ UTR reported an impact of light on recombinant protein accumulation [54], [68], others described results similar to ours [72], supporting the general concept that a decrease in translation efficiency by *psbA* 5′-UTR in darkness may be compensated by an increase in protein stability under these conditions [73], [74]. Thus, with respect to rFaeG_ntd/dsc_ yield, leaves can be harvested without concern for length or intensity of exposure to light.

### Stability of rFaeG_ntd/dsc_ in Simulated Gastrointestinal Conditions

**Figure 6:**
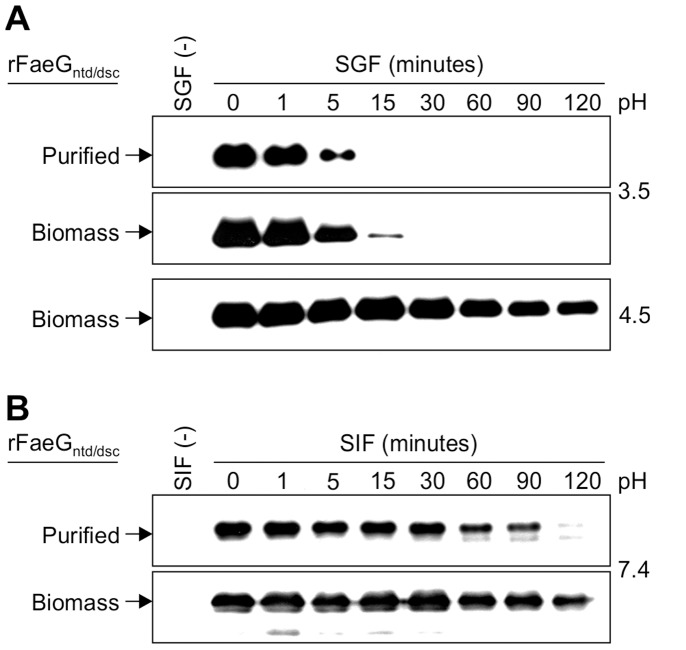
Stability of rFaeG_ntd/dsc_ under simulated gastrointestinal conditions. Time course analysis of the stability of chloroplast-expressed rFaeG_ntd/dsc_ in simulated gastric fluid (SGF; a) and simulated intestinal fluid (SIF; b). rFaeG_ntd/dsc_ was present in similar amounts either as purified protein (“Purified”) or as lyophilized and powdered transplastomic leaf tissue (“Biomass”) and was visualized by western blotting. SGF digestion of leaf biomass was done at two different pH values: pH = 3.5 and pH = 4.5. SGF and SIF fluids with no substrate [SGF (−) and SIF (−), respectively] represent negative controls. The rFaeG_ntd/dsc_ band is indicated with an arrow.

Stability of an orally-delivered ETEC vaccine in conditions present in porcine stomach and intestine is a prerequisite for successful stimulation of the mucosal immune response in the piglet gut [75], [76]. To test whether chloroplast-produced rFaeG_ntd/dsc_ would survive porcine gastrointestinal conditions, we ran *in vitro* assays in simulated piglet gastric and intestinal fl uids (SGF and SIF, respectively). In those assays we used either purified rFaeG_ntd/dsc_ protein or freeze-dried, pulverized rFaeG_ntd/dsc_-expressing leaf tissue as a substrate in a time course experiment over 2 hours. The acidity of SGF was adjusted to pH = 3.5, representing an average baseline pH in piglet stomach [76]. These SGF conditions brought about rapid degradation of the purified rFaeG_ntd/dsc_, which was undetectable after 5 minutes of digestion (Fig. 6a). Testing the rFaeG_ntd/dsc_-expressing leaf tissue as a substrate we found that addition of 0.2 g of lyophilized leaf material in 20 ml of SGF at pH = 3.5 increases the pH of the solution to pH = 4.5; this likely reflects the *in vivo* situation, where the gastric pH of fed pigs rises to 4.4 [77]. In man, the postprandial gastric pH was reported to rise up to 6.0 and then gradually drop to pH = 2.0 over a 4 h period [78]. At pH = 4.5, we found that biomass-embedded rFaeG_ntd/dsc_ was stable over the 2-hour digestion in SGF (Fig. 6a, lower panel). However, because a pH of 4.5 weakens the proteolytic function of pepsin, and to determine the survival of rFaeG_ntd/dsc_ at a pH of 3.5, the initial SGF solution was acidified to pH = 2.0 prior to addition of the leaf biomass. In this experiment, powdered lyophilized leaves were thoroughly ground in acidified SGF in a mortar and pestle, thus simulating chewing and gastric mixing. Degradation of rFaeG_ntd/dsc_ embedded within the plant tissue was slower than that of purified rFaeG_ntd/dsc_, with the protein still detectable after 15 minutes of digestion (Fig. 6a). Thus, the plant biomass matrix demonstrated a potential in delaying degradation of chloroplast-produced rFaeG_ntd/dsc_ in piglet gastric fluid, probably by providing an abundant competitive substrate in the form of endogenous plant proteins for gastric proteases. Also, the physical complexity of the plant biomass may have a “bio-encapsulating” effect and act as a preserving slow-release factor, and delaying access of gastric proteases to chloroplast-expressed rFaeG_ntd/dsc_. On the other hand, the SIF assay with both purified rFaeG_ntd/dsc_ and rFaeG_ntd/dsc_-expressing leaf biomass had very little impact on rFaeG_ntd/dsc_ protein survival (Fig. 6b). These results therefore emphasize that gastric digestion represents the limiting step for the stability of chloroplast-produced rFaeG_ntd/dsc_ inside the piglet gastrointestinal tract, and that leaf biomass could possibly serve as a shelter-vehicle to protect rFaeG_ntd/dsc_ from digestion. Since gastric fluid pH plays an important role in rFaeG_ntd/dsc_ degradation, oral administration of lyophilized leaves expressing rFaeG_ntd/dsc_ would be most effective if the vaccine is ingested upon neutralization of piglet gastric pH with a proton pump inhibitor such as rabeprazole, as was shown with *E. coli*-produced rFaeG monomers [24]. It has also been previously shown that embedding in a protective excipient improved F4 fimbriae stability against gastric acidity and proteases [79]. Therefore, it is reasonable to propose testing oral administration of rFaeG_ntd/dsc_-expressing leaf biomass, possibly coupled with neutralization of gastric pH or embedding in a protective excipient as a new vaccination strategy against F4+ ETEC infections in newly weaned piglets.

### Functional in vitro Analyses of Chloroplast-expressed rFaeG_ntd/dsc_

To test the functionality of chloroplast-produced rFaeG_ntd/dsc_, we performed an F4-specific ELISA and examined the binding of rFaeG_ntd/dsc_ to the brush borders of porcine F4R+ small intestinal villi. Additionally, we assessed the ability of rFaeG_ntd/dsc_ to competitively inhibit the attachment of F4+ ETEC to these villi.

**Figure 7:**
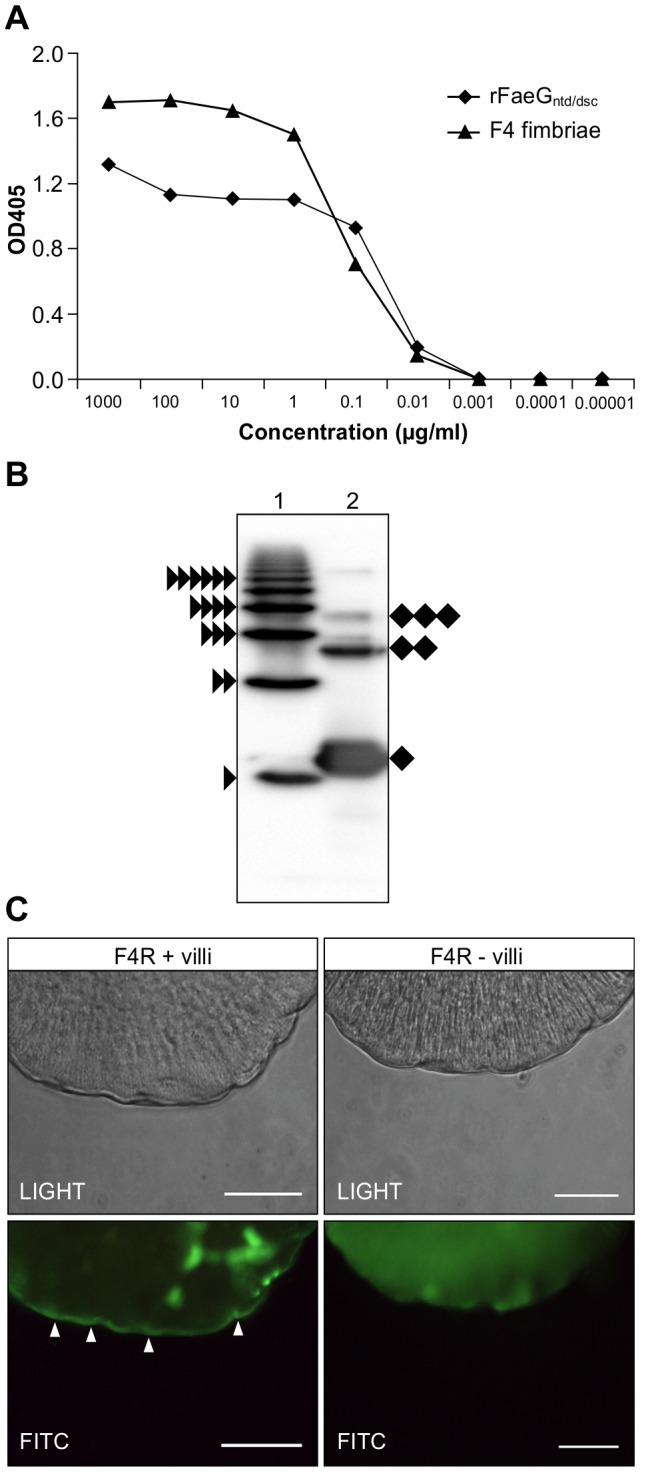
Chloroplast-produced rFaeG_ntd/dsc_ protein is recognized in F4 fimbriae-specific ELISA, partially polymerizes and specifically binds to the brush border of F4R+ small intestinal villi. (a) Both rFaeG_ntd/dsc_ and F4 fimbriae are recognized by a monoclonal anti-F4_ad_ fimbriae antibody in ELISA. (b) Purified F4 fimbriae (lane 1) and purified rFaeG_ntd/dsc_ (lane 2) were resolved under non-reducing conditions to assess polymerization. The F4 fimbriae sample displayed the formation of native FaeG polymers, number of subunits is indicated by stacked black triangles next to each band. Most of the rFaeG_ntd/dsc_ is present as monomers (denoted by black rhomb); formation of rFaeG_ntd/dsc_ dimers and trimers was also observed (two and three stacked black rhombs). (c) Adhesion of the rFaeG_ntd/dsc_ protein to the brush border of F4R+ small intestinal villi. Binding to the F4-specific receptors present on the apical surface of the epithelial cells, which line the brush border of F4R+ small intestinal villi is shown as a bright line on the edge of the sample, the result of excited FITC fluorochrome (indicated with white arrows, lower panel). rFaeG_ntd/dsc_ fails to bind to brush border of F4R− small intestinal villi. Images are representative of rFaeG_ntd/dsc_ adhesion to isolated villi of three F4R+ and two F4R− piglets. Bar: 50 µm.

Both purified F4 fimbriae and chloroplast-produced rFaeG_ntd/dsc_ were readily recognized by F4-specific rabbit serum in western blot experiments as well as by a monoclonal anti-F4 antibody ELISA (Fig. 7a). ELISA data indicated correct native conformation-like folding of the chloroplast-produced rFaeG_ntd/dsc_ subunit. Prompted by our observation that rFaeG_ntd/dsc_ dimers might be forming in transplastomic plants (Fig. 3b), we examined dimerization/polymerization of the rFaeG_ntd/dsc_ by running the purified protein under non-reducing conditions and comparing with the purified F4 fimbriae sample (Fig. 7b). The results indicate that despite the fusion of the complementary donor strand, some rFaeG_ntd/dsc_ monomers polymerize to form dimers and trimers, suggesting that donor strand exchange still occurs occasionally between rFaeG_ntd/dsc_ subunits. Worthy to notice that a higher degree of polymerization of the F4 fimbriae was correlated with a better F4-specific mucosal immunogenicity in orally-immunized piglets [80], thus, the observed partial polymerization of rFaeG_ntd/dsc_ could be beneficial to its vaccine properties if binding sites for the receptor-carbohydrates are still available in these oligomers.

These results suggested that rFaeG_ntd/dsc_ could bind to F4R and inhibit the attachment of F4+ ETEC to these receptors on the brush borders of porcine small intestinal villi similarly to F4 fimbriae [81]. This ability makes it an ideal oral subunit vaccine, since efficient F4R binding would evoke an active mucosal immune response, until neutralizing native IgA antibodies are present in the intestine. Indeed, we found that the rFaeG_ntd/dsc_ protein specifically binds to the brush borders of F4R+ villi and not to the brush borders of F4R− villi (Fig. 7c), also confirming a previous observation that the N-terminal His-tag fusion present on the rFaeG protein does not affect its interaction with F4R [24]. Although binding of the rFaeG_ntd/dsc_ protein to subepithelial cells irrespective of the F4R status of the villi was observed, we confirmed the specific binding to F4R present on the apical surface of the epithelial cells, which line the brush border of F4R+ small intestinal villi (Fig. 7c).

**Figure 8:**
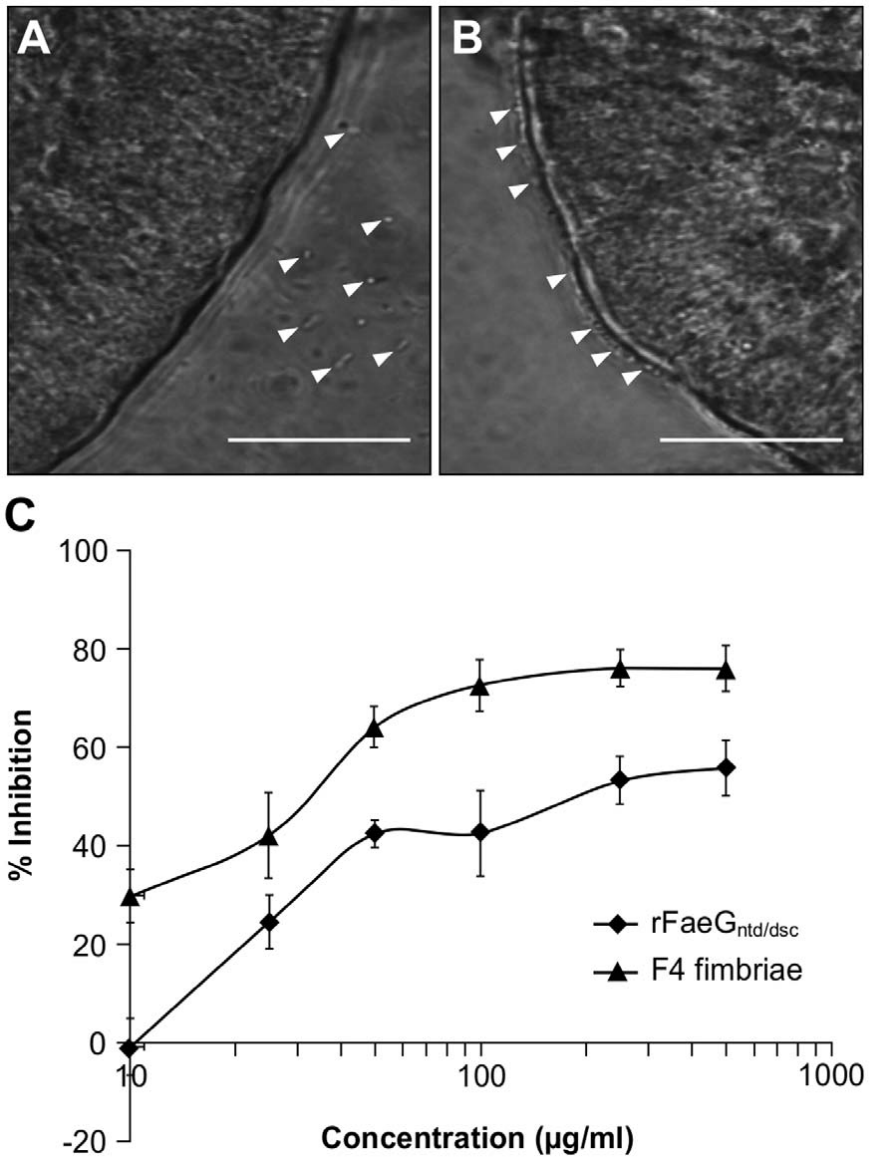
Chloroplast-produced rFaeG_ntd/dsc_ inhibits the adhesion of F4+ ETEC to porcine small intestinal villi. Adhesion of F4+ ETEC to F4R− villi (a) and F4R+ villi (b), white arrows indicate bacterial cells. Bar: 50 µm. (c) Competitive inhibition of adhesion of F4+ ETEC to porcine small intestinal villi by the rFaeG_ntd/dsc_ protein or F4 fimbriae, determined at different protein concentrations. The data represent the mean ±SE (n = 4).

To further verify the functionality of this potential subunit vaccine protein, the ability of rFaeG_ntd/dsc_ to inhibit the attachment of F4+ ETEC by competitive binding to F4R+ small intestinal villi was analyzed (Fig. 8). Chloroplast-produced rFaeG_ntd/dsc_ clearly reduced F4+ ETEC adhesion to F4R+ brush borders in a dose-dependent manner (Fig. 8c). Although rFaeG_ntd/dsc_ exhibited a similar F4R binding profile as compared to purified F4 fimbriae, a less efficient inhibition of F4+ ETEC adhesion to F4R+ villi was observed. The reduced efficiency could be due to the predominant monomeric character of the rFaeG_ntd/dsc_ protein, or to the addition of an N-terminal His-tag, but can likely be compensated by increasing the administered dose.

Cumulatively, the high level accumulation in tobacco leaves and *in vitro* characterization results of chloroplast-produced rFaeG_ntd/dsc_ suggest that this engineered recombinant adhesin could be tested as a potential oral subunit vaccine against F4+ ETEC-induced PWD in newly weaned piglets *in vivo*. The use of a high-biomass, low alkaloid tobacco cultivar accumulating gram-quantities of rFaeG_ntd/dsc_ per plant could allow simple vaccine production, which could be directly administered to animals in a lyophilized form and without extensive plant tissue processing. Given that oral administration with 2.0 mg of purified F4 fimbriae could protect F4R+ piglets against a subsequent challenge with F4+ ETEC [82], and according to our expression results of 2 mg/g fresh leaf weight, only 1 g of fresh leaf material (∼200 mg leaf dry weight) may need to be administered per piglet. However, additional studies are needed to establish the appropriate dosage of rFaeG_ntd/dsc_ for inducing protective immune response in consuming animals, since polymeric F4 fimbriae possess higher immunogenicity than refolded *E. coli*-produced rFaeG monomers [24], and since *in vivo* immunogenicity of this rFaeG_ntd/dsc_ variant has not been tested yet. Consumption of low-alkaloid tobacco at concentrations up to 30% of the diet was well tolerated by mice [83], thus, a potential need for administration of larger doses of rFaeG_ntd/dsc_-expressing tobacco leaf tissue for piglets should not raise concern. Likewise, studies focused on feasibility of feeding lyophilized rFaeG_ntd/dsc_-expressing leaf tissue to piglets are required.

### Conclusions

We report the production of transplastomic tobacco plants expressing high levels of rFaeG_ntd/dsc_, an engineered variant of the major subunit FaeG from ETEC F4 fimbriae and a potential oral vaccine candidate against porcine ETEC-induced PWD. Chloroplast-expressed rFaeG_ntd/dsc_ displays biological activity, such as *in vitro* binding to F4-specific epithelial receptors and inhibiting F4+ ETEC adhesion to porcine small intestinal villi, thus showing potential for further development and *in vivo* testing of this protein in an animal model.

## Materials and Methods

### Transient Expression of rFaeG_ntd/dsc_ in Nicotiana Benthamiana Leaves

Expression vector pJJJ109, a pCaMterX-based construct [84], carries an engineered variant F4 *rfaeG_ntd/dsc_* clone, originating from the naturally-occurring ETEC strain C1360-79 (Serotype F4_ad_; Protein Data Bank entry 3GEA; [8]. The coding sequence of the *rfaeG_ntd/dsc_* was fused at the N-terminus to the chloroplast-targeting transit peptide from pea RUBISCO small subunit. Transient expression of the rFaeG_ntd/dsc_ protein in *N. benthamiana* leaves was carried out as described in [52].

### Chloroplast Transformation Vector Construction

Details of the chloroplast transformation vector (pCT) construction can be found as Supporting Information (Methods S1). The *rfaeG_ntd/dsc_* gene was PCR-amplified from pJJJ109 with primers rFaeG*-*NheI-F: 5′-ATATGGCTAGCTGGATGACTGGTCATCACCATCACCATC-3′ and rFaeG*-*NotI-R: 5′-TACTAGCGGCCGCTTATGCAGTGATACTACCACCGATATCGAC-3′, incorporating *Nhe* I and *Not* I restriction sites (underlined) for subsequent cloning. The *rfaeG_ntd/dsc_* PCR-amplified sequence was digested with *Nhe* I and *Not* I and introduced into pre-cut pCT vector by directional cloning into the corresponding restriction sites, producing pCT-rFaeG_ntd/dsc_ (Fig. 2a).

### Generation of Transplastomic Plants and Confirmation of Homoplastomy

Transplastomic tobacco plants (cv. I 64) were obtained by the biolistic method [85], [86]. Following 3 regeneration rounds on selective medium containing 500 µg/ml spectinomycin, homoplastomy of all the clones was confirmed by Southern blot analysis. Three µg of plant total DNA (Qiagen DNeasy Plant Mini kit, Qiagen, GmbH), were completely digested with *Rsr* II enzyme, separated on 0.8% agarose gel and transferred onto Hybond-N+ membrane (Amersham Biosciences, UK). DIG-labelled probe was amplified with primers Probe-F 5′-CACCACGGCTCCTCTCTTCTCG-3′ and Probe-R 5′-TTCCTACGGGGTGGAGATGATGG-3′ using PCR DIG Probe Synthesis kit (Roche Diagnostics, GmbH) and pPF as template. Hybridization of the probe was carried out at 50°C overnight. Five high stringency washes (100 mL of 2XSSC +0.1% SDS at 23°C – twice; 100 mL of 0.5XSSC +0.1% SDS at 68°C – three times) were performed, followed by 30 min blocking at 42°C and 30 min of antibody binding with 3 subsequent washes. Detection was carried out by autoradiography.

### Recombinant Protein Extraction and Quantification

Proteins were extracted by homogenizing leaf tissue in liquid N_2_ in a Tissuelyser (Qiagen, GmbH) then vortexing with 3 to 10 volumes of Extraction Buffer 1 (EB1) (50 mM Na-Acetate, 15 mM CaCl_2_, pH 4.9) or EB2 (Phosphate Buffered Saline [PBS]: 137 mM NaCl, 2.7 mM KCl, 10 mM Na_2_HPO_4_, 2 mM KH_2_PO_4_ pH = 7.5, 1% Tween-20, 1 mM EDTA, 2% [w/v] PVPP), both supplemented with 1% phenylmethylsulfonyl fluoride (PMSF) and 0.1% leupeptin. EB1 was used for the characterization of rFaeG_ntd/dsc_ accumulation in Figure 3 only. Total proteins were sampled from the crude homogenate, and total soluble proteins were sampled after centrifugation for 10 minutes at 14000×g. To assess the amount of rFaeG_ntd/dsc_ trapped in the pellet of insoluble plant material after centrifugation of EB2-extracted leaf tissue, the pellet was re-dispersed in an equal volume of EB2 by vortexing, centrifuged, and sampled. TSP concentration was measured using the Bradford assay [87] and BSA as a standard.

Purification of rFaeG_ntd/dsc_ from crude leaf extract was performed with a 2-step procedure. First, the rFaeG_ntd/dsc_-containing plant extract was clarified by acidification to pH = 2.0 with concentrated HCl causing most plant endogenous proteins to precipitate. Subsequent to centrifugation, the pH of the resulting supernatant was adjusted to neutral (pH = 7.4) with KOH. Recombinant rFaeG_ntd/dsc_ was then purified by IMAC on a 1 ml His-Trap™ (GE Healthcare, USA) column. Quantification of purified rFaeG_ntd/dsc_ was carried out by densitometry analysis of serial dilutions of rFaeG_ntd/dsc_ of a stained SDS-PAGE gel using TotalLab TL100 software (Nonlinear Inc., Durham, USA) and known amounts of BSA.

To assess levels of rFaeG_ntd/dsc_ protein accumulation in transplastomic leaves, protein immunoblots were detected with anti-FaeG rabbit serum [26], horseradish peroxidase-conjugated goat anti-rabbit IgG (1∶5000, Bio-Rad Laboratories, USA), and ECL (Amersham ECL Western Blotting Systems, GE Healthcare, USA), followed by autoradiography rFaeG_ntd/dsc_ was quantified by densitometry with TotalLab TL100 software (Nonlinear Inc., Durham, USA) using known amounts of purified rFaeG_ntd/dsc_ protein to generate the standard curve (*R* ^2^ = 0.998).

### SGF and SIF Experiments

Simulated gastric fluid (SGF) and simulated intestinal fluid (SIF) analyses were conducted as previously described [21], with a few modifications. Freeze-dried transgenic tobacco leaves (0.2 g) were homogenized in 20 ml of either SGF (pH = 2 or pH = 3.5) or SIF (pH = 7.4) using a mortar and pestle. The emulsions were incubated at 37°C and samples were taken at various time points. These were subsequently neutralized and analyzed by SDS-PAGE. The SGF and SIF were prepared as described by [88]–[91].

### Animals and Samples for in vitro Studies

Sampling of villi from piglets was performed according to the local animal welfare regulations and approved by the ethics committee of the Faculty of Veterinary Medicine, Ghent University. Pigs (Large White×Belgian Landrace) were 6 to 7 weeks old when euthanized. To assess the capacity of rFaeG_ntd/dsc_ to adhere to F4R present on the brush border of porcine small intestinal villous enterocytes, intestinal villi were isolated as described by [81]. Subsequently, the villi were scraped off with glass slides, washed 4 times in Krebs-Henseleit buffer and stored at −20°C.

### F4 Fimbriae-specific ELISA

F4_ad_ fimbriae were purified from the *E. coli* strain H56 (08:K87:F4_ad+_) as described by [81]. A 96-well plate (Maxisorp immunoplates, NUNC, Roskilde, Denmark) was coated with an F4_ad_-specific mAb (CVI, Lelystad, The Netherlands), blocked overnight at 4°C in PBS +0.2% Tween®80 and washed with PBS +0.2% Tween®20 (TPBS). Serial dilutions of the rFaeG_ntd/dsc_ protein and purified F4_ad_ fimbriae were added to the coated plates, incubated for 1 h at 37°C and washed with TPBS. Next, the plates were incubated with heat-inactivated F4-specific porcine serum for 1 h at 37°C, washed and finally incubated with an optimal concentration of HRP-conjugated anti-porcine IgG for 1 h at 37°C. Following several wash steps, an ABTS solution was added and the optical density was measured at 405 nm (OD_405_) after 15 and 30 min incubation at 37°C. To remove background signals, a cut-off value was calculated as followed: cut-off value  =  mean OD_405_ 0 µg/ml F4_ad_ fimbriae +2*sd. This cut-off value was subtracted from OD_405_ values.

### Brush Border Binding Assay

To analyze the epithelial binding capacity of rFaeG_ntd/dsc_, both F4R+ and F4R− villi were washed and the FcR were blocked by incubating the villi for 30 min at RT while shaking with PBS +5% heat-inactivated goat serum. Subsequently, the villi were incubated with 500 µg/ml rFaeG_ntd/dsc_, heat-inactivated F4-specific rabbit serum and FITC-conjugated goat anti-rabbit IgG F(ab’)_2_ (Sigma) for 45 min at RT while shaking. Villi were mounted on glass slides and the rFaeG_ntd/dsc_ binding was analyzed with a fluorescence microscope at 488 nm wavelength (Leica Microsystems). Images were captured with a digital camera from Scion Corporation and processed with ImageJ software.

### In vitro Villous Adhesion and Inhibition Assay

The F4R status of the isolated villi was determined in an *in vitro* villous adhesion assay by incubating the isolated small intestinal villi with 4×10^8^ F4_ad_+E. coli (strain H56) at room temperature (RT) for 45 min while gently shaking as previously described [18]. The adhesion of the bacteria was evaluated by counting the number of bound bacteria along 50 µm villous brush border at 20 randomly selected places with a phase-contrast microscope at a magnification of 400X. Adhesion of >5 bacteria per 250 µm villous brush border is considered as positive [92].

The F4R binding capacity of rFaeG_ntd/dsc_ was assessed in an *in vitro* villous adhesion inhibition assay [80]. Villi of four F4R+ and two F4R− piglets were incubated with rFaeG_ntd/dsc_ or purified F4_ad_ fimbriae for 45 min at RT while gently shaking. Subsequently, F4_ad_+E. coli were added and the adhesion of the bacteria to the villi was analyzed as described above. The percentage of inhibition of bacterial adhesion was calculated for each rFaeG_ntd/dsc_ or F4_ad_ fimbriae concentration by comparing with mock-treated villi as follows: % inhibition  = 100–((x/y)*100); where x =  number of bacteria/250 µm brush border at given concentration of rFaeG_ntd/dsc_ or F4 fimbriae; and y =  number of bacteria/250 µm brush border at 0 µg/ml rFaeG or F4.

## Supporting Information

Methods S1 Construction of the chloroplast transformation vector pCT.
(DOC)
