# Supplementary material for: Production of a Subunit Vaccine Candidate against Porcine Post-Weaning Diarrhea in High-Biomass Transplastomic Tobacco
Source: PLoS One. 2012 Aug 3;7(8):e42405. doi: 10.1371/journal.pone.0042405 (PMC3411772; doi:10.1371/journal.pone.0042405)
Supplement: Methods S1 — Construction of the chloroplast transformation vector pCT. (DOC) [file pone.0042405.s001.doc]

**Supporting Information**

**Production of a subunit vaccine candidate against porcine post-weaning diarrhea in high-biomass transplastomic tobacco**

Igor Kolotilin, Angelo Kaldis, Bert Devriendt, Jussi Joensuu, Eric Cox and Rima Menassa.

**Methods S1**

*Chloroplast transformation vector pCT construction*

Integration of the cassette into the tobacco plastome was designed to occur between the *trnI* *(tRNA-isoleucine)* and *trnA (tRNA-alanine)* genes. Sequences of cis-regulatory elements in the transformation construct were chemically-synthesized and assembled into the designed cassette by a series of restriction endonuclease digestion/ligation reactions. For that, a fragment of the tobacco plastome (nucleotides 104,084 – 106,578; Accession Number NC001879) was PCR-amplified with primers Flanx-F: 5’-CTAACCCCAAAAACCCGTCCTCAGT-3’ and Flanx-R: 5’-cccatttcgctcgccgctactac-3’, cut by *EcoR* I, treated with Mung Bean nuclease (NEB, MA, USA) to create blunt ends and ligated into the *Pvu* II-cut, alkaline phosphatase-treated pUC19 vector, creating the Plastome Flanx vector (pPF). All the DNA amplification procedures were done using Expand High Fidelity PCR System (Roche Diagnostics, GmbH), with subsequent sequencing. The tobacco chloroplast promoter of the *psbA* gene (P*psbA*; *N. tabacum* chloroplast genome NC001879, complementary nucleotides 1598-to-1737) along with its 5' UTR was used for expression of the *rfaeG_ntd/dsc_* gene; expression of the *aadA* gene relied on read-through transcription from the endogenous upstream *rrn* promoter. The *aadA* gene, encoding aminoglycoside 3' adenylyltransferase was PCR-amplified from plasmid pXW-rbcL-AAD, kindly provided by Dr. Shengwu Ma. The 3' end of the intercistronic expression element (IEE, *N. tabacum* chloroplast genome NC001879, nucleotides 77030-to-77095), facilitating efficient processing of polycistronic mRNAs , was fused to the Shine-Dalgarno sequence from the 5' UTR of bacteriophage T7 gene *10* (GAAGGAG) and integrated upstream of the *aadA* gene. The 3' ends of the *faeG* and the *aadA* coding sequences were fused to 3' UTR regions of the *rbcL* (T*rbcL*) and *psbC* (T*psbC*) plastid genes from White poplar (*Populus alba*, chloroplast genome NC008235; nucleotides 56790-to-57022 and 34875-to-35052), respectively. The transformation cassette was introduced into the *Nsi* I site in the *trnI-trnA* spacer of the pPF, creating the pCT vector.

References:

1. Svab Z, Hajdukiewicz P, Maliga P (1990) Stable transformation of plastids in higher plants. Proc Natl Acad Sci USA 87: 8526-8530.

2. Svab Z, Maliga P (1993) High-frequency plastid transformation in tobacco by selection for a chimeric aadA gene. Proc Natl Acad Sci USA 90: 913-917.

3. Zhou F, Karcher D, Bock R (2007) Identification of a plastid intercistronic expression element (IEE) facilitating the expression of stable translatable monocistronic mRNAs from operons. Plant Journal 52: 961-972.
